# Theaflavin 3, 3′-Digallate Delays Ovarian Aging by Improving Oocyte Quality and Regulating Granulosa Cell Function

**DOI:** 10.1155/2021/7064179

**Published:** 2021-12-08

**Authors:** Jiahuan He, Guidong Yao, Qina He, Tongwei Zhang, Huiying Fan, Yucheng Bai, Junya Zhang, Guang Yang, Ziwen Xu, Jingyi Hu, Yingpu Sun

**Affiliations:** ^1^Center for Reproductive Medicine, The First Affiliated Hospital of Zhengzhou University, Zhengzhou, China; ^2^Henan Key Laboratory of Reproduction and Genetics, The First Affiliated Hospital of Zhengzhou University, Zhengzhou, China

## Abstract

Ovarian aging refers to the gradual decline of ovarian function with increasing physiological age, manifested as decreased ovarian reserve, elevated aging-related markers, and reduced oocyte quality. With a declining female fertility and a growing aging population, it is urgent to delay ovarian aging to maintain fertility and improve the life quality of women. Theaflavin 3, 3′-digallate (TF3) is a naturally bioactive polyphenol compound extracted from black tea, and its antioxidant properties play an important role in maintaining human health and delaying aging; however, the effects of TF3 on female reproduction and ovarian function are not yet clear. Here, we show that TF3 can preserve primordial follicle pool, partially restore the estrous cycle, and increase the offspring number of aged mice. Meanwhile, TF3 gavage increased the number of oocytes retrieved, decreased the level of reactive oxygen species, increased the level of glutathione, and decreased the abnormal rate of oocyte spindle after ovulation induction. Moreover, TF3 inhibited human granulosa cell apoptosis and improved their antioxidative stress ability. High-throughput sequencing and small-molecule-targeted pharmacological prediction show that TF3 affects multiple pathways and gene expression levels, mainly involved in reproductive and developmental processes. It may also affect cellular function by targeting mTOR to regulate the autophagic pathway, thereby delaying the process of ovarian aging. This study shows that TF3 can be used as a potential dietary supplement to protect ovary function from aging and thereby improving the life quality of advanced-age women.

## 1. Introduction

Ovarian aging is defined as a gradual decline in ovarian function with age. Female fertility generally begins to decline after the age of 30 and declines more significantly after 35. The general postponement of women's childbearing age nowadays makes reproductive aging a common fertility concern [1]. The clinical manifestations of fertility decline include decreased ovarian reserve, female endocrine hormone disorders, and irregular menstrual cycles [2], which are mainly related to a series of ovarian function downregulation indicators such as increased secretion of aging inflammatory substances in the ovarian microenvironment, abnormal granulosa cell number and function, and decreased oocyte quality [3–5].

Accumulation of reactive oxygen species (ROS) is closely associated with organ aging [6]. Studies have reported that oxidative stress damage caused by excessive accumulation of ROS in the ovary leads to impaired granulosa cell function and decreased oocyte quality, which causes reduced follicle number and quality as well as endocrine abnormalities, ultimately leading to ovarian aging [7]. In women undergoing *in vitro* fertilization embryo transfer (IVF-ET), the ROS content in both oocytes and granulosa cells is significantly higher in older patients than that in younger patients, with reduced antioxidant levels [8]. The high levels of ROS in follicular fluid may be associated with poor embryo quality [9]. This suggests that excessive ROS in the ovaries of older women is an important factor leading to decreased ovarian function and adverse pregnancy outcomes. Reducing ROS levels in the ovary could play a pivotal role in improving female ovarian function and prolonging female fertility.

Tea polyphenols are important natural antioxidant compounds derived from food. Theaflavin extracted from black tea fermentation is an antioxidant class with natural bioactivities, mainly including theaflavin (TF1), theaflavin-3-gallate (TF2A), theaflavin-3′-gallate (TF2B), and theaflavin 3, 3′-digallate (TF3) [10]. Among these four monomers, the proportion of TF3 components is highest and has the greatest antioxidant effect [11]. Previous studies on tea polyphenols have focused on the green tea extract epigallocatechin gallate (EGCG), which is a powerful antioxidant. EGCG supplementation can inhibit the transition from primordial follicle to growing follicle, which is essential for prolonging the follicular growth phase, reducing oocyte apoptosis, and protecting female fertility [12]. However, in recent years, it was discovered that tea polyphenol TF3 in fermented black tea even possesses stronger antioxidant activity than EGCG [13]. TF3 shows antioxidant function by modulating an organism's biological enzyme activity system to eliminate free radicals [11] and preventing hydroxyl radical-induced DNA damage by scavenging ROS [14]. In a mouse model of nonalcoholic fatty liver disease and primary hepatocytes, the theaflavin family attenuated inflammatory responses and reduced hepatocyte apoptosis through antioxidant pathways [15].

In a double-blind, randomized controlled study in humans, oral supplementation with TF3 significantly reduced inflammatory factors and oxidative stress injury due to high-intensity exercise [16]. Others have suggested that TF3 is a noncompetitive adenosine triphosphate (ATP) synthase inhibitor that reduces energy metabolism by directly binding to ATPase without inducing or even decreasing superoxide production [17]. Current research on TF3 in the female reproductive system has focused on a possible antitumor ability, and multiple studies have indicated that TF3 can protect female fertility by selectively inhibiting ovarian cancer cell proliferation [18, 19]. Nevertheless, the functional role of TF3 in the regulation of ovarian aging remains unclear.

In this study, we first investigated the role of TF3 supplementation in improving fertility and ovarian function in aged mice and further studied the effects of TF3 on oocyte quality, embryonic developmental potential, and granulosa cell function. In addition, the potential molecular mechanism of TF3 in regulating granulosa cell function was studied by sequencing analysis and small molecule compound-protein simulation docking. These results demonstrate that TF3 is a therapeutically promising polyphenol compound and plays an important role in protecting ovarian function from aging.

## 2. Materials and Methods

### 2.1. Ethical Approval

This study was approved by the Scientific Research and Clinical Trial Ethics Committee of the First Affiliated Hospital of Zhengzhou University.

### 2.2. Animals and Experiment Design

All CD-1 mice used in this study were purchased from Vital River (Vital River, Beijing, China) and housed in the Henan Animal Experimental Center with a 12 h dark/light cycle and at a room temperature of 22–25°C. Nine-month-old, aged female mice were randomly divided into two groups: old TF3 gavage experimental (O-TF3) and old control (O-Ctrl). The O-TF3 group mice were fed with TF3 (Biopurify, Chengdu, China) at 30 mg/kg every other day, and the O-Ctrl group mice were fed with an equal volume of normal saline every other day; both groups were, respectively, treated for 90 days.

Eight-week-old adult CD-1 female mice served as young controls (Y-Ctrl). For mating tests, 8-week-old adult male mice were cohabited with three groups at a ratio of 1 : 2 and housed separately after 1 week. The number of offspring was statistically analyzed.

### 2.3. Estrous Cycle Monitoring

At the end of the gavage period, the estrous cycle of mice was examined using the smear method. The vaginal orifice was exposed, and vaginal secretions were collected with a small cotton swab and smeared on a glass slide. The samples were fixed with 95% ethanol for 5 min, dried, stained with hematoxylin for 15 min, and then rinsed with water for 5 min, dehydrated with 95% ethanol for 3 min, and then repeatedly rinsed and stained. The cell morphology was observed under a light microscope. Estrous cycle monitoring was based on a previous study [20]. In the diestrus, there were a few shrunken, nucleated epithelial cells and occasional keratinocytes. During proestrus, epithelial cells were significantly reduced, with more basophilic cells or cells that lost the ability to be stained. In estrus, almost all the enucleated keratinocytes were stained red without white blood cells; during metestrus, nucleated epithelial cells and leukocytosis were present.

### 2.4. *In Vitro* Fertilization and Immunofluorescence Staining of Oocytes

At the end of treatment, mice were intraperitoneally injected with 10 IU pregnant mare serum gonadotropin (PMSG; Solarbio, Beijing, China); after 48 h, the mice were intraperitoneally injected with 10 IU human chorionic gonadotropin (hCG; Livzon, Zhuhai, China). Fourteen hours later, the mice were sacrificed by cervical dislocation, and cumulus oocyte complexes (COCs) were obtained at the tubal enlargement and washed in handling medium G-MOPS™ Plus (Vitrolife, Goteborg, Sweden) preheated at 37°C and then transferred to G-IVF™ Plus medium (Vitrolife) for culture prior to insemination. Adult male sperm were obtained 1 h in advance for capacitation in G-IVF™ Plus droplets, and the capacitated sperm were added to droplets containing COCs for insemination. Fertilized oocytes were removed 4 h after insemination and continued to be cultured and observed in G-1™ Plus (Vitrolife). The culture medium was changed to G-2™ Plus (Vitrolife) droplets on the third day after insemination to continue culturing until blastocyst development. The following formulae were used:

(1) $$\begin{array}{c} \text{Fertilization rate }\left(\%\right)=\frac{\text{number of fertilized zygotes}}{\text{recovered oocytes}}\times 100\%,\\ \text{Blastocyst formation rate }\left(\%\right)=\frac{\text{number of blastocysts}}{\text{cleaved embryos}}\times 100\%. \end{array}$$

COCs used for immunofluorescence staining were digested with hyaluronidase (Vitrolife) to remove granulosa cells around oocytes, and subsequent experiments were performed after the granulosa cells were decontaminated. ROS (Beyotime, Shanghai, China), JC1 (Beyotime), and Glutathione (GSH) Detection Reagent (Invitrogen, Carlsbad, CA, USA) kits were used for staining according to the manufacturer's protocols. Oocytes were stained for 30 min at 37°C, eluted three times using phosphate-buffered saline (PBS) with 0.1% Triton X-100 (PBST), and then imaged using a confocal laser scanning microscope (Zeiss LSM 700, Oberkochen, Germany).

For spindle staining experiments, oocytes after granulosa cell removal were fixed in 4% paraformaldehyde (PFA) for 1 h at room temperature, after which they were transferred to 0.5% Triton X-100 permeabilization solution formulated using 4% PFA for 30 min at room temperature. At the end of permeabilization, three washes were performed using PBST, blocked using 5% bovine serum albumin (BSA) for 1 h at room temperature, and then incubated with *α*-tubulin-FITC (1 : 200, Thermo Fisher Scientific, Waltham, MA, USA) and diluted in 5% BSA overnight at 4°C. After incubation, oocytes were transferred to an antifluorescent quencher with 4′,6-diamidino-2-phenylindole (DAPI) dye (Cell Signaling Technology, Danvers, MA, USA) for mounting and photography after three washes in PBST. Blastocysts were stained using DAPI in blastocyst cell counting experiments, imaged under a confocal laser scanning microscope, and counted according to the number of nuclei.

### 2.5. Serum Hormone Assay

Mouse serum follicle-stimulating hormone (FSH), estradiol (E_2_), and progesterone (P4) levels were measured using a chemiluminescence immunoassay kit (Roche Diagnostics, Basel, Switzerland) on a Roche Diagnostics Cobas 6000 analyzer.

### 2.6. Ovarian Follicle Grading and Counting

After the mice were sacrificed by cervical dislocation, the ovaries were removed and transferred into 4% PFA, embedded in paraffin, and then sectioned serially at a thickness of 5 *μ*m; every fifth slide was stained with hematoxylin and eosin (HE). Follicular grading and counting methods were based on a previous publication [21].

### 2.7. Immunohistochemical Staining

Mouse ovaries were fixed in 4% PFA, then embedded in paraffin, and sectioned to 5 *μ*m thick section obtained. The sections with the largest cross sections were used for staining. Slides were baked at 65°C for 1 h, deparaffinized in xylene, soaked in distilled water after graded ethanol addition into the water along with antigen retrieval, and incubated in 3% hydrogen peroxide solution for 10 min at room temperature. After that, slides were washed three times with PBS and incubated with 5% BSA for 10 min. After pouring off the liquid, overnight incubation was performed at 4°C with antibodies against mammalian target of rapamycin (mTOR) (1 : 1000, Abcam, Cambridge, UK) or P16 (1 : 1000, Abcam). For negative controls, serum was used instead of primary antibody. After overnight incubation, the primary antibody was discarded, the slides were washed three times with PBS, and horseradish peroxidase secondary antibody (Goat Anti-Rabbit IgG H&L, 1 : 5000, Abcam) was added for 1 h at 37°C. After incubation, the slides were washed in PBS three times, and DAB chromogen was added dropwise. After color development was complete, the slides were washed thrice in PBS and counterstained for 20 s in hematoxylin. The slide was then rinsed with tap water for 3 min, mounted using neutral gum, and imaged under a light microscope.

### 2.8. Masson's Trichrome Staining

Paraffin-embedded tissue was processed as described above and stained using the Masson Stain Kit (Servicebio, Wuhan, China). Mouse ovary sections were dewaxed in xylene in turn and soaked in distilled water with gradient ethanol addition; then, the sections were washed using distilled water and soaked in Masson A dye solution overnight. After overnight culture, the sections were rinsed with tap water, and then, they were immersed in the dye solution mixed with Masson B and Masson C in equal proportion for 1 min. After that, the sections were rinsed with distilled water for 1 min, soaked in 1% hydrochloric acid alcohol for 1 min, then rinsed with tap water, and then immersed in Masson D dye solution for 6 min followed by a final rinse in tap water. After that, the sections were immersed in Masson E for 1 min, placed in Masson F staining solution for 30 s after turning slightly dry, rinsed with 1% glacial acetic acid for 1 min, dehydrated twice with absolute ethanol, and soaked in fresh absolute ethanol for 5 min. Finally, they were immersed in xylene for transparentizing for 5 min, mounted in neutral gum, and imaged.

### 2.9. Cell Culture

Human primary granulosa cells (pGCs) were collected from the follicular fluid of patients undergoing IVF-assisted pregnancy at reproductive center of the First Affiliated Hospital of Zhengzhou University, and the specific methods were based on previous publications [22].

### 2.10. RNA Extraction and Real-Time Polymerase Chain Reaction (PCR)

After treatment, the cells were first washed once with precooled PBS, and the cells were lysed by adding the corresponding volume of TRIzol (Invitrogen); 200 *μ*l chloroform was added per 1 ml of TRIzol, and the tube was then vigorously shaken for 15 s, placed at room temperature for 10 min, and then centrifuged the sample at 12,000 rpm for 15 min at low temperature. The upper aqueous phase was harvested, followed by addition of 200 *μ*l isopropanol, vigorous shaking for 15 s, and allowing to stand at room temperature for 10 min, and then centrifuged the sample at 12,000 rpm for 15 min at low temperature; the supernatant was discarded, followed by washing twice with 75% ethanol and addition of an appropriate amount of RNase-free double-distilled H_2_O for dissolution to determine RNA concentration and quality. Qualified RNA was reversely transcribed using the iScript™ cDNA Synthesis Kit (Bio-Rad, Hercules, CA, USA), and reversely transcribed cDNA was detected by fluorescence quantitative PCR using the SYBR® Green Master Mix Kit (Bio-Rad) with a QuantStudio 12K Flex instrument (Applied Biosystems, Foster City, CA, USA). All primer sequences are shown in Supplementary Table S1.

### 2.11. Western Blotting

Treated granulosa cells were washed using prechilled PBS, and 100 *μ*l radioimmunoprecipitation assay buffer (Solarbio) lysate was added to each well and lysed on ice for 15 min. The lysed cells were transferred to a centrifuge tube and centrifuged at 12,000 rpm for 15 min at 4°C, and then, the supernatant protein was collected for quantification and treated with sodium dodecyl loading buffer at 100°C for 5 min before being stored at -80°C until future use. Electrophoresis was performed using a precast gel (Genscript, Nanjing, China) and transferred to a 0.45 *μ*m PVDF membrane under semidry transfer at 25 mA for 14 min. The membranes were blocked using 5% skimmed milk for 1 h at room temperature, and then, the membranes were incubated overnight at 4°C with primary antibodies. At the end of the primary antibody incubation, the corresponding secondary antibody was added for 1 h at room temperature after elution three times with TBST. At the end of secondary antibody incubation, the membranes were washed in TBST three times, and bands were developed using an enhanced chemiluminescence detection system (Bio-Rad). The primary antibodies used were as follows: Bcl-2 (1 : 1000, Abcam), BAX (1 : 1000, Abcam), Caspase-3 (1 : 1000, Abcam), *γ*H2AX (1 : 1000, Abcam), nuclear factor- (NF-) *κ*B (1 : 1000, Cell Signaling Technology); mTOR (1 : 1000, Abcam); *β*-actin (1 : 5000, Bioworld, Bloomington, MN, USA); and GAPDH (1 : 5000, Abcam). The mouse and rabbit secondary IgG antibodies (1 : 5000) were both from Abcam. Beta-actin and GAPDH were used as internal controls.

### 2.12. Apoptosis and ROS Analysis by Flow Cytometry

Granulosa cells were routinely cultured in 6-well plates for 24 h. After starving for 24 h, the cells were cultured with different concentrations of TF3 or controls for another 48 h and then washed thrice with PBS. Apoptosis detection was performed using the Annexin V-FITC/PI Apoptosis Kit (KeyGEN BioTECH, Nanjing, China). A Reactive Oxygen Species Assay Kit (Beyotime) was used to evaluate ROS levels in granulosa cells. The samples were analyzed on a BD C6 Flow Cytometer (BD Biosciences, Franklin Lakes, NJ, USA).

### 2.13. *β*-Galactosidase Staining

Senescence staining analysis was performed using the Senescence *β*-Galactosidase Staining Kit (Beyotime) according to the kit instructions. Briefly, pGCs and KGN cell lines were seeded in 6-well plates at a density of 2 × 10^5^ cells/well and routinely cultured for 24 h. Serum-free culture medium was starved for 24 h, followed by different concentrations of TF3 or control culture for 48 h. At the end of treatment, the cells were washed with PBS three times, fixed in a fixative at room temperature for 15 min, stained with staining solution at 37°C overnight, and then photographed for analysis. For periovarian adipose tissue, the samples were rinsed in PBS three times, fixed in the fixative at room temperature for 15 min, and then stained with the staining solution in the kit at 37°C overnight prior to imaging.

### 2.14. Immunofluorescence Staining of Cells

The treated granulosa cells were fixed using 4% PFA for 30 min at room temperature and then permeabilized with PBST for 30 min at room temperature. At the end of permeabilization, 5% BSA was used to block for 1 h at room temperature and *γ*H2AX antibody (1 : 1000, Abcam) was added for overnight incubation at 4°C. Rabbit IgG secondary antibody (1 : 5000, Abcam) was added after three PBST washes for a 60 min incubation at room temperature. At the end of secondary antibody incubation, the samples were washed three times in PBST and mounted with antifluorescence quencher containing DAPI. Images were analyzed on a laser scanning confocal microscope.

### 2.15. Cell Viability Analysis

Cell proliferation viability was detected and analyzed using the cell counting kit-8 (CCK-8; Dojindo, Tokyo, Japan). Briefly, pGCs were routinely cultured in 24-well plates at 1 × 10^5^ seeds per well for 24 h, starved for another 24 h, and then treated with different doses of TF3 for 96 h. After treatment, the culture medium was changed to 300 *μ*l of DMEM containing 10% CCK-8 solution, and the culture was continued for 2 h. The luminosity value at 450 nm was detected on a Varioskan Flash microplate reader (Thermo Fisher Scientific).

### 2.16. RNA Sequencing and Analysis

Human pGCs were routinely cultured for 24 h and then starved for 24 h. The culture medium was changed to the one containing 10 *μ*M TF3, or the control medium was used for a total of 6 h incubation. At the end of the treatment, cellular RNA was collected, cDNA libraries were constructed, and transcriptome PE150 sequencing was performed using the Illumina NovaSeq platform (Illumina, San Diego, CA, USA). Target prediction and functional analysis were performed on 454 selected differential genes using Metascape Online [23]. The GSEA was performed using the OmicStudio tools at https://www.omicstudio.cn/tool.

### 2.17. Statistical Analysis

All experiments were performed in triplicate, and results are presented as mean ± SEM. Statistical analyses were performed using *t*-tests. A value of *p* < 0.05 was considered to indicate statistical significance.

## 3. Results

### 3.1. TF3 Improves Ovarian Function and Increases Litter Size in Aged Mice

**Figure 1:**
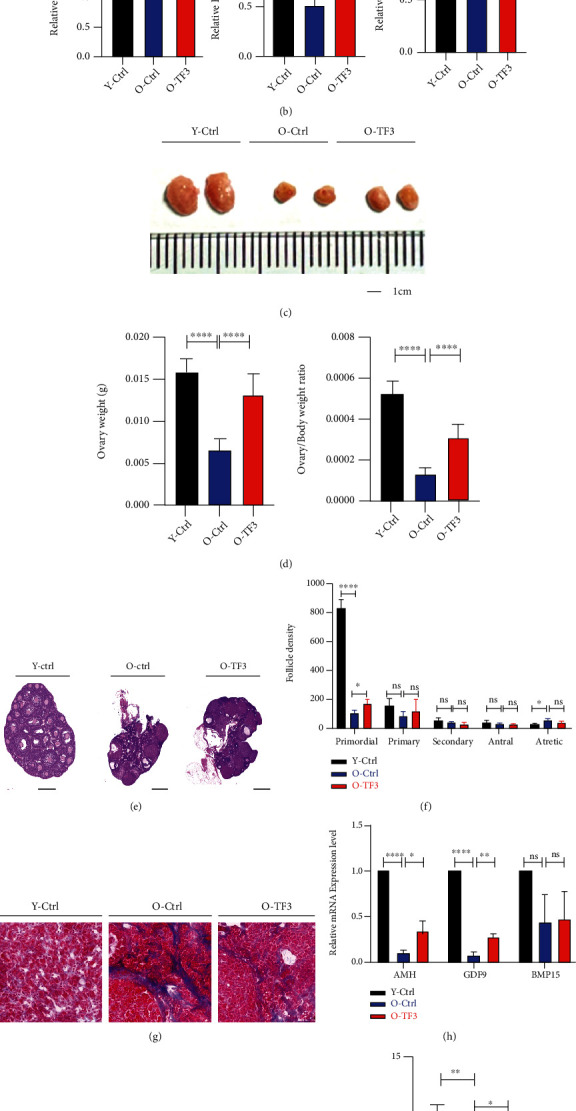
TF3 promotes ovarian function maintenance in aged mice. Vaginal cells were obtained from aged mice treated with intragastric administration (TF3 or control) for 90 days and 8-week-old young control mice without intragastric administration for 14 days. Estrous cycle was monitored and analyzed by HE staining, expressed as a line graph (a). P denotes metestrus, E denotes estrus, and M/D denotes diestrus and proestrus. Serum FSH, E_2_, and P4 secretion levels were measured by enzyme-linked immunosorbent assays (b). After gavage administration, the ovaries were photographed to compare the change of ovarian volume (bar = 1 cm) (c). The body and ovary weights of mice in each group after treatment were obtained, and the ovary weight and ovary/body weight ratio (d) of mice in each group were compared and analyzed. HE staining was performed on the largest cross sections to observe and analyze ovarian and follicular development (bar = 500 *μ*m) (e). Follicle count (f) after HE staining of serial ovarian sections and Masson staining of frozen ovarian sections were performed to analyze ovarian fibrosis status (bar = 20 *μ*m) (g). Total RNA was extracted from mouse ovaries, and the expression levels (h) of ovarian reserve markers AMH, GDF9, and BMP15 mRNA were analyzed by real-time PCR. GAPDH was the internal reference gene. At the end of gavage treatment, the mice were naturally mated with adult male mice, and the litter size (i) was recorded. Young Ctrl (Y-Ctrl): 8-week-old CD-1 mice; old Ctrl (O-Ctrl): 9-month-old CD-1 mice treated with saline for 90 days; old+TF3(O-TF3): 9-month-old CD-1 mice treated with 30 mg/kg/day TF3 for 90 days. ^∗^*p* < 0.05, ^∗∗^*p* < 0.01, ^∗∗∗^*p* < 0.001, and ^∗∗∗∗^*p* < 0.0001.

To explore the effect of TF3 on ovarian function, aged mice were given gavage administration of TF3 (30 mg/kg) every two days for 90 days, and aged mice in the control group were given the same volume of saline. At the end of treatment, estrous cycles were monitored for 14 consecutive days, and the results revealed that the estrous cycle of the old TF3 group increased compared with that of the old control group, although it was still prolonged compared with the young control group (Figure 1(a)). Serum FSH level in the old control group was significantly higher than that in the young control group (*p* < 0.01) and decreased after TF3 treatment (*p* < 0.05); estradiol and progesterone levels were not significantly different between the groups (*p* > 0.05) (Figure 1(b)).

The ovarian volume of aged mice was significantly increased after TF3 gavage treatment (Figures 1(c) and 1(e)), and the ovarian weight and ovarian/body weight ratio were significantly higher than those of old controls (*p* < 0.001) (Figure 1(d)). Hematoxylin and eosin staining of the treated ovarian sections was performed to analyze the effect of TF3 on follicular development, and the results revealed that more follicles remained on the ovaries in the TF3-treated group than the old control group (Figure 1(e)). The number of primordial follicles was also significantly increased (*p* < 0.05) (Figure 1(f)). More interestingly, the degree of ovarian interstitial fibrosis decreased in TF3-treated aged mice compared with old controls (Figure 1(g)). In addition, the mRNA expression level analysis of ovarian reserve markers showed that the expression levels of AMH, BMP15, and GDF9 in old control mice were significantly lesser than those in the young controls (*p* < 0.05); nonetheless, TF3 treatment restored the expression level of AMH and GDF9 (*p* < 0.05) (Figure 1(h)).

To investigate the effect of TF3 on mouse fertility, we performed natural fertility assays in different groups of mice. The litter size of young control mice was the highest after natural mating, that of old control mice was significantly decreased (*p* < 0.01), and the litter size of mice treated with TF3 gavage was higher than that of the old control group (*p* < 0.05) (Figures 1(i) and 1(j)).

### 3.2. TF3 Downregulates the Expression Level of Ovarian Aging-Related Genes

**Figure 2:**
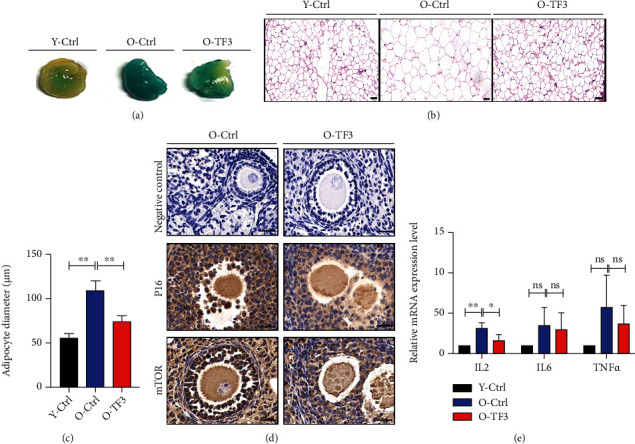
Effect of TF3 on the expression of marker genes of aging in mice. After treatment, adipose tissue around the ovary was stained for the aging marker galactosidase (a) and HE to statistically analyze lipid droplet size (b, c) (bar = 50 *μ*m). Immunohistochemical staining of ovarian tissue sections showed expression of P16 and mTOR protein, with negative control (d) on the left side (bar = 20 *μ*m). Total RNA was extracted from the ovaries and reversely transcribed, and the relative expression levels of *IL-2*, *IL-6*, and *TNFα* were analyzed using real-time PCR, with GAPDH as the internal reference gene (e). ^∗^*p* < 0.05, ^∗∗^*p* < 0.01, and ^∗∗∗∗^*p* < 0.0001.

The above findings indicate that TF3 can improve ovarian function and increase litter size in aged mice, which seems to indicate that TF3 helps delay ovarian aging. The aging of adipose tissue around the ovary is closely related to ovarian function and aging. In this study, beta-galactosidase staining was first performed on adipose tissue adjacent to the ovary, and the results showed adipose tissue in the aged control group was darker, which became lighter after TF3 treatment, indicating that TF3 treatment reduced the aging level of adipose tissue around the ovary (Figure 2(a)). Hematoxylin and eosin staining analysis of periovarian adipose tissue sections showed that the size of lipid droplets in ovarian adipose tissue of the aged control group was significantly increased compared with that of the young group, and TF3 treatment significantly decreased lipid droplet size (*p* < 0.01) (Figures 2(b) and 2(c)). Immunohistochemical results revealed reduced expression of mTOR and p16 in the ovarian tissue of aged mice treated with TF3 (Figure 2(d)). RNA was extracted from the ovaries of treated mice to analyze mRNA expression levels of inflammatory marker genes interleukin- (IL-) 2, *IL-6*, and tumor necrosis factor-alpha (*TNFα*). TF3 treatment significantly decreased IL-2 mRNA levels (*p* < 0.01), and IL-6 and TNF*α* tended to decrease after TF3 treatment but not significantly (*p* > 0.05) (Figure 2(e)).

### 3.3. TF3 Improves Oocyte Quality in Aged Mice

**Figure 3:**
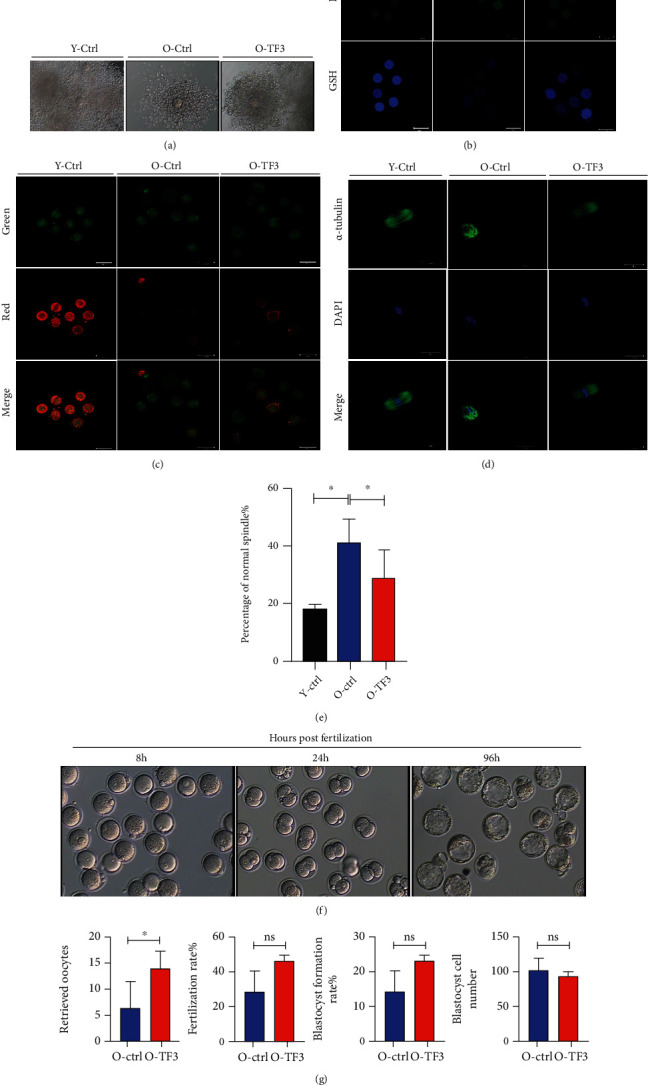
TF3 slows down the age-related decline in mouse oocyte quality. Mice in each treatment group were intraperitoneally injected with 10 IU PMSG and, after 48 h of treatment, injected with 10 IU hCG, and COCs were obtained from the ampulla of the fallopian tube 14 h later to analyze the effect of TF3 on COC morphology (a). The oxidative stress status of oocytes was analyzed by ROS and GSH staining after removing granulosa cells from COCs obtained from each treatment group (b). JC1 staining was performed to analyze oocyte mitochondrial membrane potential; red (polymers) and green (monomers) represent higher and lower oocyte mitochondrial membrane potential, respectively (c). The effect of TF3 on oocyte spindle morphology was analyzed by staining oocytes with *α*-tubulin antibody (green) and DAPI (blue); the right panel shows statistical analysis of abnormal spindle morphology and the ratio (d, e). COCs obtained by ovarian hyperstimulation were inseminated *in vitro* to analyze the effects of TF3 on oocyte retrieval, fertilization rate, blastocyst formation rate, and blastocyst cell number in aged mice (f, g). ^∗^*p* < 0.05.

Oocyte quality directly reflects ovarian function. In view of this, mice in each group were subjected to ovulation induction, and the harvested COCs were used in subsequent experiments. Compared with young control mice, the granulosa cells around COCs in aged control mice were relatively loosely arranged and scarce, while the number of granulosa cell layers around COCs was tightly arranged after TF3 treatment (Figure 3(a)). To further assess the effect of TF3 treatment on mouse oocyte quality, granulosa cells were removed from the obtained COCs for immunofluorescence staining. The results showed that the ROS level of oocytes in the aged control group was higher than that in the young control group, and levels of the antioxidant GSH were lower than those in the young control group. After TF3 treatment, the ROS level of oocytes in the aged mice decreased, and GSH expression increased (Figure 3(b)). Analysis of oocytes stained for the mitochondrial membrane potential marker JC1 revealed that young control mice had higher oocyte mitochondrial membrane potential, mostly in the form of JC1 polymers (red fluorescence). Aged control mice had poorer oocyte mitochondrial function and lower membrane potential, mostly in the form of JC1 monomers (green fluorescence), and TF3-treated aged mice had partially recovered mitochondrial function, and more JC1 polymers were present in the cytoplasm (Figure 3(c)). In addition, *α*-tubulin-stained spindles of mouse oocytes showed that most of the spindle arrangements in the young control group were normal, and the spindle fibers were tightly regulated and continuous, and the nuclear arrangement was tight. Furthermore, the ratio of abnormal spindle of oocytes in the aged control group was significantly increased (*p* < 0.05), which manifested as loose spindle breaks with scattered nuclear morphology. The ratio of abnormal spindles significantly decreased in the aged mice after TF3 treatment (*p* < 0.05) (Figures 3(d) and 3(e)).

To investigate the effects of different treatments on mouse embryo development, mouse oocytes were obtained after ovulation induction for *in vitro* fertilization experiments (Figure 3(f)). The number of oocytes retrieved in TF3-treated aged mice was significantly higher than that in the aged control mice (*p* < 0.05) (Figure 3(g)). In addition, although the fertilization rate and blastocyst formation rate after *in vitro* fertilization in TF3-treated aged mice were slightly increased, they were not significantly different (*p* > 0.05). Furthermore, the numbers of blastocyst cells were not significantly different between the two groups (*p* > 0.05) (Figure 3(g)).

### 3.4. TF3 Inhibits Apoptosis of Cultured pGCs

**Figure 4:**
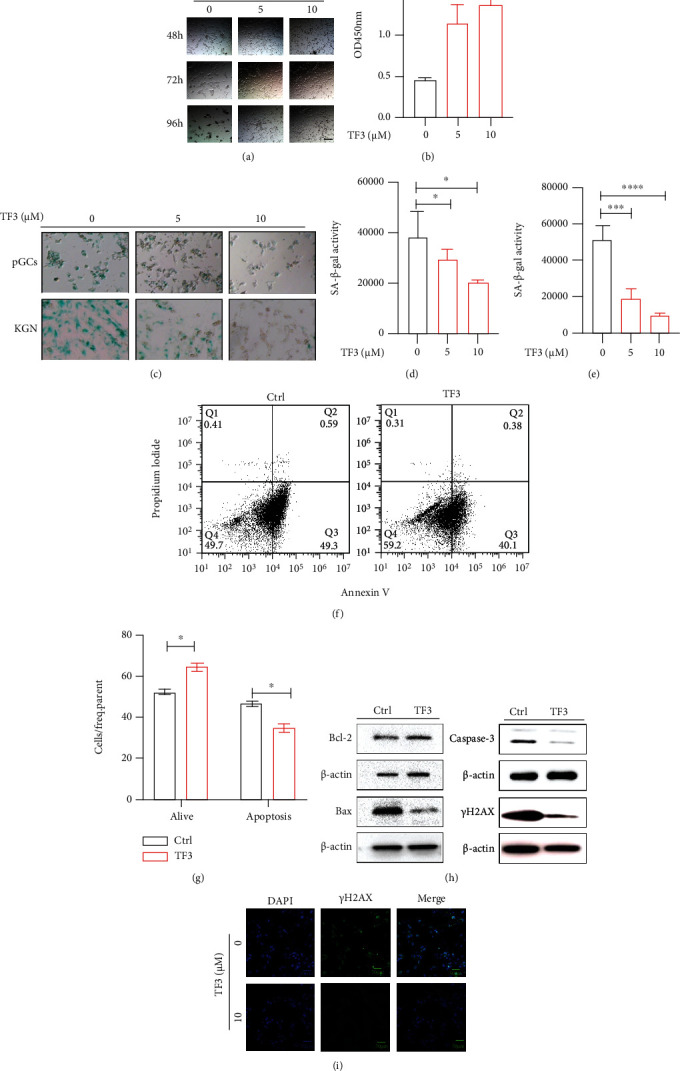
TF3 inhibits apoptosis of granulosa cells cultured *in vitro*. Granulosa cells were routinely cultured and treated with different concentrations of TF3 (0, 5, and 10 *μ*M) with the time to start TF3 addition set at 0 h, bar = 50 *μ*m (a). CCK8 cell viability assay kits were used to detect the effect of TF3 on cell viability after 120 h of culture (b). Human pGCs and KGN granulosa cell lines were cultured to analyze the effect of TF3 on beta-galactosidase expression, bar = 50 *μ*m (c). Quantitative statistical analysis of beta-galactosidase staining in pGCs (d) and KGN (e) cells was performed using ImageJ software. Flow cytometry was performed to analyze the effect of TF3 (10 *μ*M) on pGC apoptosis (f), and the ratios of apoptosis of different types of cells were statistically analyzed (g). Total protein was extracted from pGCs treated with TF3/control, and the expression levels of apoptosis-related proteins Bcl-2, BAX, caspase-3, and *γ*H2AX were analyzed by western blotting (h). Immunofluorescence staining was performed to analyze the expression of the apoptotic marker *γ*H2AX, bar = 50 *μ*m (i). ^∗^*p* < 0.05, ^∗∗∗^*p* < 0.001, and ^∗∗∗∗^*p* < 0.0001.

Follicular granulosa cells are closely related to ovarian function. To explore the potential regulatory mechanism of TF3 on ovarian function, we investigated the effect of TF3 on granulosa cell apoptosis. In an *in vitro* culture model of human pGCs, the results revealed that TF3 was beneficial to maintain healthy pGC morphology, and the number of adherent cells after 96 h of culture was significantly greater compared with the control group (Figure 4(a)). The CCK-8 cell viability assay analysis also showed that TF3 could significantly resist pGC apoptosis levels (Figure 4(b)). Staining for the aging marker galactosidase after 48 h of culture of pGCs and KGN granulosa cell lines revealed that TF3 treatment could significantly reduce the expression level of beta-galactosidase and delay cellular aging (*p* < 0.05) (Figures 4(c)–4(e)). The flow cytometry results showed that the proportion of pGC apoptosis after 48 h of TF3 treatment was significantly decreased (*p* < 0.05) (Figures 4(f) and 4(g)). Inhibition of apoptosis by TF3 in cultured pGCs may be related to the upregulation of Bcl-2/BAX ratio by TF3 and downregulation of the protein expression levels of BAX, caspase-3, and *γ*H2AX (Figure 4(h)). The quantitative results are shown in supplementary materials (Supplementary Figure S1). The results of fluorescence staining for *γ*H2AX, a marker of DNA damage, also indicated that TF3-treated pGCs had significantly reduced *γ*H2AX fluorescence (Figure 4(i)).

### 3.5. TF3 Improves the Antioxidative Ability of Granulosa Cells Cultured *In Vitro*

**Figure 5:**
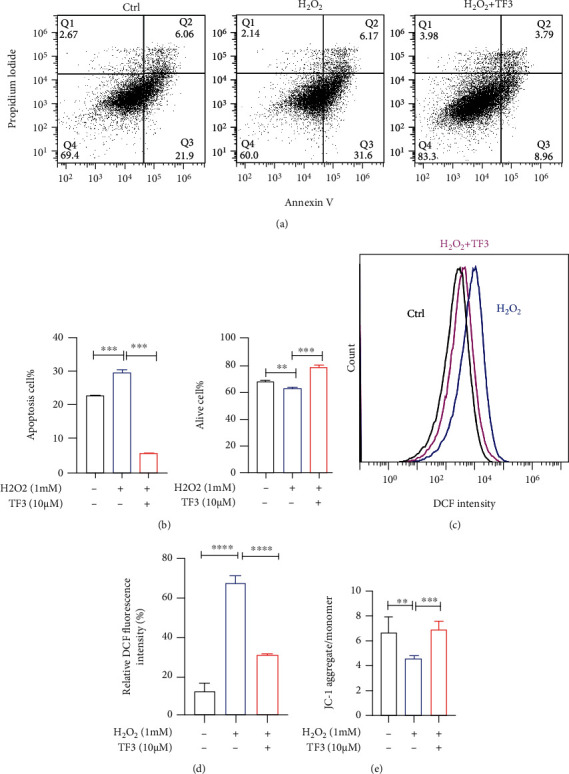
TF3 alleviates the damaging effect of oxidative stress on granulosa cells. pGCs were routinely cultured for 24 h and then starved and cultured in serum-free culture medium for 24 h. Cells were treated with TF3 (10 *μ*M) or control for 24 h and then treated with H_2_O_2_ (1 mM) or an equal volume of pure water control for another 6 h to generate a pGC oxidative stress injury model. After treatment, apoptosis under anticell stress conditions was analyzed by flow cytometry (a), and the results were statistically analyzed (b). An oxidative stress model was used to analyze the effect of TF3 on ROS levels in granulosa cells (c), and the results were statistically analyzed (d). TF3 resistance to H_2_O_2_-induced decrease in mitochondrial membrane potential (e) was analyzed by detecting intracellular mitochondrial membrane potential with JC1. ^∗^*p* < 0.05, ^∗∗^*p* < 0.01, ^∗∗∗^*p* < 0.001, and ^∗∗∗∗^*p* < 0.0001.

To further investigate the role of TF3 in granulosa cells, we constructed a H_2_O_2_-treated pGC oxidative stress model and used flow cytometry to analyze the ability of TF3 to counteract cellular oxidative stress. The results showed that H_2_O_2_ significantly increased the proportion of apoptosis (*p* < 0.001), which was attenuated by TF3 treatment (*p* < 0.001) (Figures 5(a) and 5(b)). Analysis of intracellular ROS levels revealed that TF3 could also significantly reduce the H_2_O_2_-induced increase in cellular ROS levels (*p* < 0.01) (Figures 5(c) and 5(d)). The intracellular JC1 mitochondrial membrane potential assay analysis confirmed that TF3 could significantly upregulate the decrease in mitochondrial membrane potential caused by H_2_O_2_ (Figure 5(e)).

### 3.6. TF3 May Regulate Ovarian Function by Affecting the Autophagy Pathway

**Figure 6:**
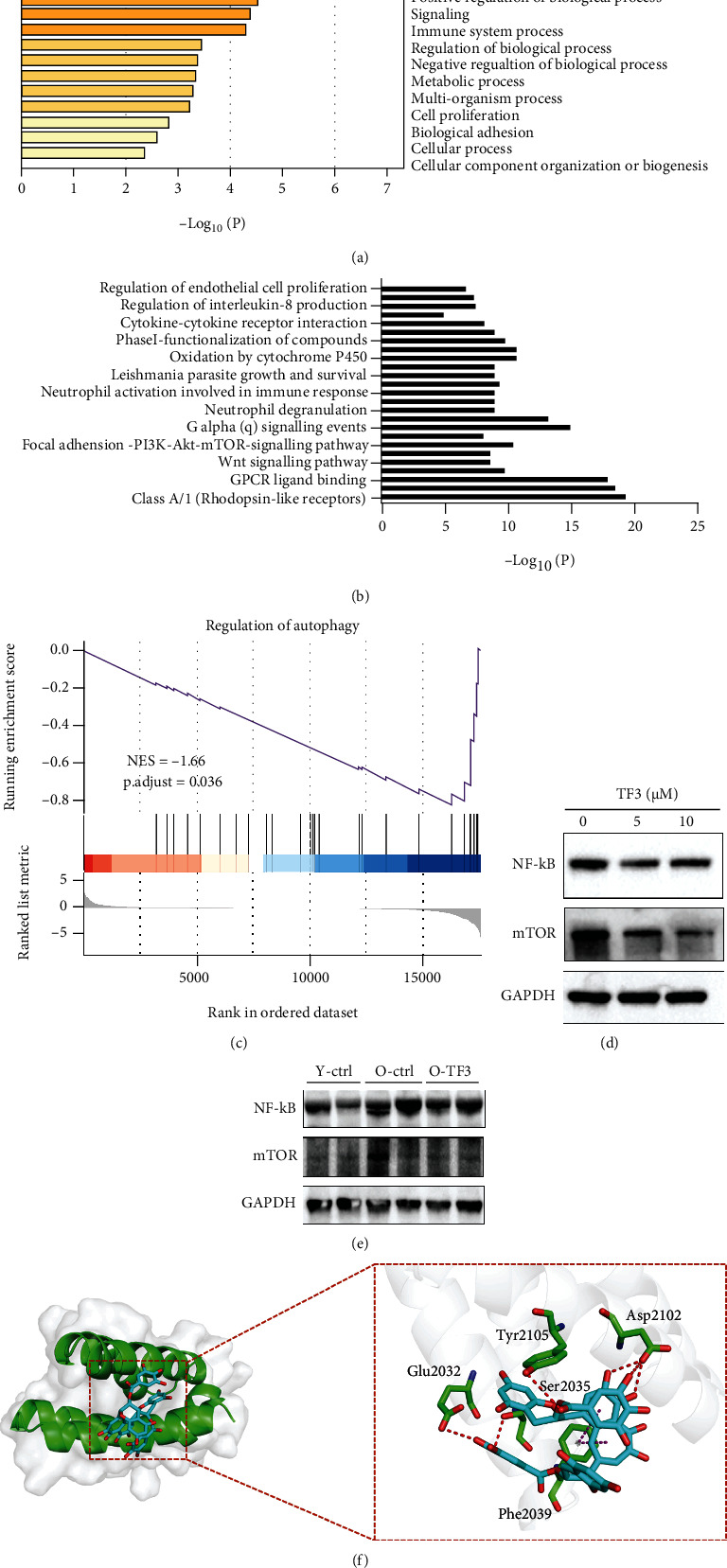
TF3 may affect granulosa cell function by regulating autophagy through the mTOR pathway. pGCs were routinely cultured for 24 h and then starved in a serum-free culture medium for 24 h. TF3 (10 *μ*M) or control treatment was given for 6 h. Cells were collected for transcriptome sequencing, and differentially expressed genes were subjected to GO analysis related to cell biological processes (a) and MCODE analysis related to signaling pathways (b). GSEA algorithm enrichment of all genes and calculation of enrichment scores were performed to find the key pathways affected by TF3 (c). Granulosa cells after TF3 treatment were collected, proteins were extracted, and the effect of TF3 on related proteins was analyzed by western blotting (d). Ovarian tissues were collected from all three groups, and proteins were extracted to analyze the effects of TF3 on NF-*κ*B and mTOR protein levels (e). GAPDH was the internal reference protein. The molecular docking mode of TF3 to mTOR protein was predicted using AutoDock Vina software to map the action mode of TF3-5WBH, with the blue part indicating TF3 and the red box indicating the docking site (f).

To further explore the potential action pathway of TF3 in regulating granulosa cell function, whole transcriptome sequencing analysis was performed on pGCs after TF3 treatment. A total of 454 genes had significantly altered the expression after TF3 treatment (*p* < 0.05) (Supplementary Table S2). The Gene Ontology (GO) analysis of differentially expressed genes indicated that TF3 in granulosa cells mainly affected the regulation of reproductive and developmental processes (Figure 6(a)). Analysis of the involved signaling pathways suggested that TF3 might be involved in the regulation of reproductive and developmental processes through the Class A/1(Rhodopsin-like receptors), GPCR ligand binding, Wnt signaling pathway, and PI3K-AKT-mTOR clusters (Figure 6(b)). Based on further analysis of key signaling pathways and significantly differential genes, we hypothesize that the mTOR pathway and its downstream associated molecular regulatory networks may be regulated by TF3. We performed enrichment analysis of the differentially expressed genes involved by using the gene set enrichment analysis (GSEA) algorithm, and the results showed that TF3 treatment could significantly affect autophagy function (*p* < 0.05) (Figure 6(c)). This was validated in granulosa cells and mouse ovaries; the results revealed that expression of the granulosa cell autophagy-related proteins mTOR and NF-*κ*B decreased after TF3 treatment, suggesting that TF3 may scavenge ROS-damaged proteins and damaged mitochondria by activating the autophagy pathway (Figure 6(d)). The quantitative results are shown in supplementary materials (Supplementary Figure S2). NF-*κ*B and mTOR protein expression levels were increased in the ovarian tissue of aged control mice compared with young control mice, while they were decreased in the ovaries of aged mice after TF3 treatment (Figure 6(e)). The quantitative results are shown in supplementary materials (Supplementary Figure S3). Furthermore, we used the AutoDock Vina method to perform semiflexible docking of TF3 to FRB domain of mTOR and selected the optimal conformation of affinity (Figure 6(f)). The affinity binding energy of TF3 to FRB domain of mTOR (5WBH) protein was -7.7 kcal/mol; six hydrogen bonds could be formed with amino acid residues Asp2102, Glu2032, Ser2015, and Tyr2105 of the mTOR protein, and two *π*–*π* interactions could be formed with Phe2039. The molecule also formed hydrophobic interactions with amino acid residues Phe2039 and Tyr2105. TF3 was predicted to affect mTOR to form a more stable complex through hydrogen bonding and hydrophobic interactions.

## 4. Discussion

The natural tea polyphenol TF3 is a small molecule compound that has become increasingly popular as a dietary supplement in clinical practice; such compounds are characterized by multiple targets with naturally low toxicity or even no toxicity [24]. In this study, intragastric administration of TF3 in aged mice significantly delayed ovarian aging, as shown by maintaining ovarian reserve, shortening estrous cycle, and enlarging litter size. Further studies revealed that TF3 treatment significantly downregulated oocyte ROS levels and abnormal spindle rates, which in turn improved oocyte quality. Our analyses of ovarian granulosa cells showed that TF3 improved the antioxidative ability of granulosa cells and decreased apoptosis, which may be related to the targeting of TF3 to bind to mTOR FRB domain and regulate the autophagy pathway.

Female reproductive performance decreases with age, manifested as the continuous depletion of follicle pools and irregular menstrual cycles; these changes are accompanied by increased FSH and decreased AMH, and excessive FSH levels will lead to follicular overgrowth, thereby exacerbating follicle pool depletion [25]. The number of primordial follicles in the ovaries of women with regular menstruation is 10 times higher than the primordial follicles in those with irregular menstruation, and the rate of follicular depletion dramatically increases during the final stages of the female reproductive life (perimenopausal period) [26]. In addition, the ability to secrete hormones and the response to pituitary hormones are important manifestations of ovarian function, and excessive FSH levels also indicate poor follicular growth [27]. In this study, we found that aged mice supplemented with TF3 had significantly lower FSH levels than the aged control group, suggesting that TF3 can improve follicular development in aged mice, which is consistent with our ovarian follicle counts. Women with endocrine disorders have reduced quality of life because of hot flashes, night sweats, sleep disorders, osteoporosis, and even depression [28, 29]. Our experiments showed that TF3 gavage treatment of 9-month-old mice was equivalent to intervention in the early perimenopausal period of humans, and the results revealed that TF3 was beneficial to maintain the follicle pool and improve ovarian function. This treatment partially restored the estrous cycle of mice, suggesting that TF3 also has a potential role in improving the quality of life of women.

The condition of periovarian adipose tissue is closely related to ovarian function; moderate adipose tissue plays a protective role, but excessive adipose tissue secretes proinflammatory factors that cause cellular senescence, which in turn leads to decreased quality of oocytes and granulosa cells and accelerates ovarian aging [30]. TF3 downregulates the expression of inflammatory factors secreted by adipose tissue [31], and a human randomized controlled trial showed that oral theaflavin administration for 10 weeks significantly reduced subcutaneous fat content and increased skeletal muscle percentage [32]. Our results showed significantly larger lipid droplet area in the periovarian adipose tissue of aged mice than in the young controls, and TF3 supplementation significantly reduced the lipid droplet area, which was consistent with previous literature reports. Beta-galactosidase staining also showed that TF3 could significantly delay ovarian adipose tissue aging in aged mice.

Overactivation of the primordial follicle pool causes persistent loss of primordial follicles, which leads to the development of premature ovarian failure (POF) [33]. The incidence of POF is approximately 1% in women and represents accelerated aging of the female reproductive system before the age of 40 years [34]. The use of chemotherapeutic drugs such as cisplatin can also lead to POF, thereby decreasing ovarian function [35]. It is important to prevent excessive activation of the follicular pool, thereby maintaining the ovarian reserve and prolonging female reproduction. Administering TF3 to aged mice preserved the primordial follicle pool and increased the litter size. These findings suggest that TF3 slows down excessive activation of the follicle pool, which is important for improving and prolonging female fertility.

The autophagy-lysosomal system is an important method to remove damaged organelles and plays a pivotal role in both targeted degradation therapy for various diseases [36] and the regulation of ovarian aging [37]. Physiologically, the oxidative stress system maintains homeostasis, and when ROS levels are slightly increased, the autophagy system is activated to scavenge them. When age-related ROS accumulation becomes excessive and autophagic activity decreases with age [38], the scavenging ability of ROS also decreases, at which time the damage caused by excessive ROS will lead to aggravated aging and chronic injury. Therefore, increasing the autophagy activity is beneficial to delay the aging processes [39] [40].

In the follicular microenvironment, granulosa cells and oocytes interact to regulate the process of follicular development. Granulosa cells can directly affect oocyte quality through gap junctions, and this can lead to decreased oocyte quality when oxidative stress is exacerbated in the granulosa cells [41]. Normal granulosa cell function is therefore essential for oocyte development [42]. The *in vitro* culture model of pGCs purified from human follicles is widely used in reproductive research [43, 44], but the viability and status of pGCs also gradually decreased with longer culture time; thus, it is a potential aging cell model. We found that TF3 could delay granulosa cell apoptosis *in vitro* and significantly reduce ROS-induced apoptosis and oxidative stress injury in the H_2_O_2_-induced oxidative stress model. Beta-galactosidase staining was performed on pGCs, KGN cells, and mouse periovarian adipose tissues cultured *in vitro*; the results suggested that TF3 treatment could delay the senescence of granulosa cells and periovarian adipose tissue. This indicates that TF3 can promote lysosomal degradation at the cellular and organism levels to facilitate the removal of senescent cells and harmful substances and effectively slow down the aging process.

Studies on oocytes derived from TF3-treated aged mice revealed that TF3 can reduce ROS levels, increase GSH levels, attenuate the decrease of mitochondrial membrane potential in oocytes, and improve oocyte quality. Presumably, TF3 can reduce damage to the ovary caused by ROS overaccumulation through scavenging ROS and increasing antioxidant enzyme activities. Tea polyphenols have been found to partially restore meiotic abnormalities, DNA damage, and apoptosis in porcine oocytes after cisplatin exposure [45], which is consistent with our findings that TF3 can significantly reduce the ratio of abnormal spindles in oocytes from aged mice. In addition, it was reported that *in vitro* maturation of bovine oocytes and the addition of certain concentrations of tea polyphenols to the *in vitro* culture medium can improve the conception rate [46]. Tea polyphenols such as TF3 seem to have a positive effect on oocyte maturation and embryo development *in vitro*.

Some researchers have found that TF3 can act directly on ATP-binding enzymes, thereby affecting their structural activity and inhibiting ATP turnover and production [17]. Polyphenolic compounds can also regulate the aging processes by targeting mTOR [47]. These studies seem to suggest that TF3 may protect ovarian function by regulating energy metabolism pathways. To clarify the role of TF3 in ovarian follicle development, we performed RNA sequencing of granulosa cells after TF3 treatment and performed deep GSEA of the sequencing results, which showed that TF3 mainly affected reproductive and developmental processes by regulating autophagy and pathways such as PI3K-AKT-mTOR.

Autophagy reduces the death of healthy cells and has an important role in maintaining homeostasis. In addition to its ability to remove potential prototoxins, autophagy improves mitochondrial function and helps recovery from acute cellular injury with damaged mitochondria; in addition, it can help avoid apoptosis or necrosis [48]. During aging, oxidative stress and increased inflammation can cause DNA damage and accumulation of I*κ*B kinases, leading to increased expression of mTOR and NF-*κ*B, thereby inhibiting autophagy and further exacerbating aging [49, 50]. Polyphenolic compounds can mimic caloric restriction to induce autophagy [51], mainly by increasing AMP-activated protein kinase to decrease mTOR and induce autophagy [38]. Our sequencing results confirmed that TF3 mainly regulates granulosa cell function by affecting the autophagy pathway, and further analysis showed that the natural small molecule compound TF3 may act on mTOR FRB domain to exert its regulatory effect.

## 5. Conclusions

**Figure 7:**
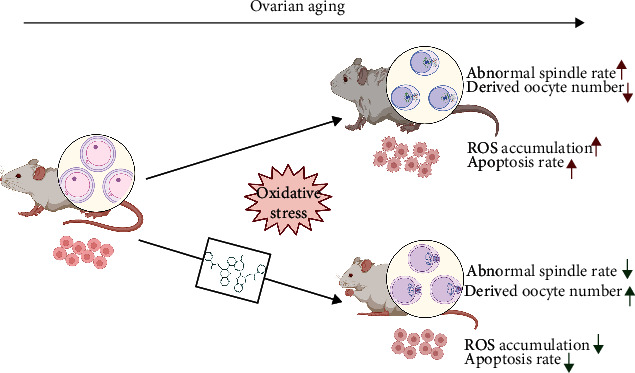
The mechanism model of TF3 working on ovarian function. This chart illustrates a possible mechanism model by which TF3 regulates oocyte quality and granulosa cell function during the process of ovarian aging. This work is created with http://BioRender.com with the permission to publish.

*In vitro* and *in vivo* mouse model experiments revealed that TF3 can effectively slow down the process of ovarian aging and improve ovarian function in aged mice by improving oocyte quality and granulosa cell function (Figure 7). As a natural polyphenolic compound, TF3 could regulate mTOR to reduce ovarian metabolism to maintain the ovarian reserve, reduce the accumulation of ROS due to ATP overproduction, improve ovarian autophagy to remove aging substances, reduce ROS damage to oocytes and granulosa cells, delay female ovarian aging, and prolong female fertility.

## Data Availability

The data will be available in the supplementary materials.

## Supplementary Materials

Supplementary 1 Supplementary Table S1: primers used in the study.

Supplementary 2 Supplementary Table S2: differentially expressed genes treated by TF3 in primary granulosa cells.

Supplementary 3 Supplementary Figure S1: the statistical analysis of apoptosis protein expression in different groups. Supplementary Figure S2: statistical analysis of NF-*κ*B and mTOR protein expression in different groups. Supplementary Figure S3: statistical analysis of NF-*κ*B and mTOR protein expression in different groups.
