# Supplementary material for: Theaflavin 3, 3′-Digallate Delays Ovarian Aging by Improving Oocyte Quality and Regulating Granulosa Cell Function
Source: Oxid Med Cell Longev. 2021 Dec 8;2021:7064179. doi: 10.1155/2021/7064179 (PMC8674650; doi:10.1155/2021/7064179)
Supplement: Supplementary 3 — Supplementary Figure S1: the statistical analysis of apoptosis protein expression in different groups. Supplementary Figure S2: statistical analysis of NF-κB and mTOR protein expression in different groups. Supplementary Figure S3: statistical analysis of NF-κB and mTOR protein expression in different groups. [file 7064179.f3.pdf]

Supplementary materials

Supplementary figures

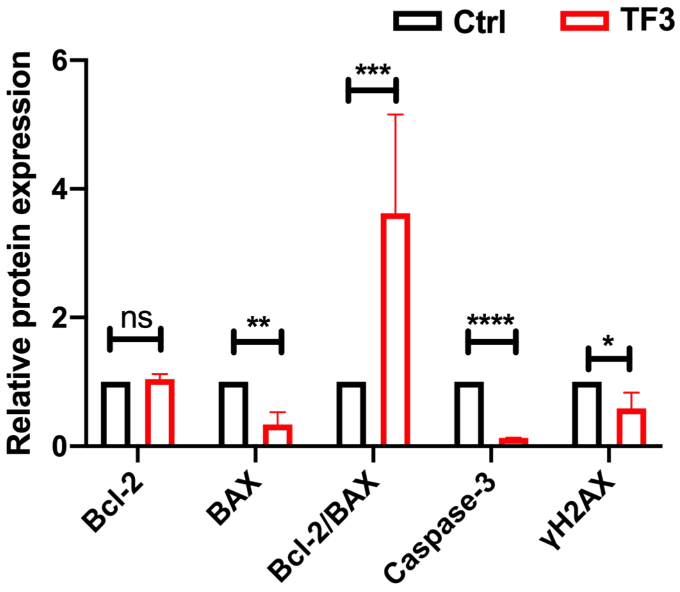

Supplementary Figure S1. The Statistical analysis of apoptosis protein expression in different groups. Granulosa cells were routinely cultured for 24 h after seeding, starved in serum-free culture medium for 24 h, and then treated with TF3 (10 μM) or control. The apoptosis related proteins were measured, and the statistical analysis was performed. ns, no significant difference; *p<0.05, **p<0.01, ***p<0.001, and ****p<0.0001.

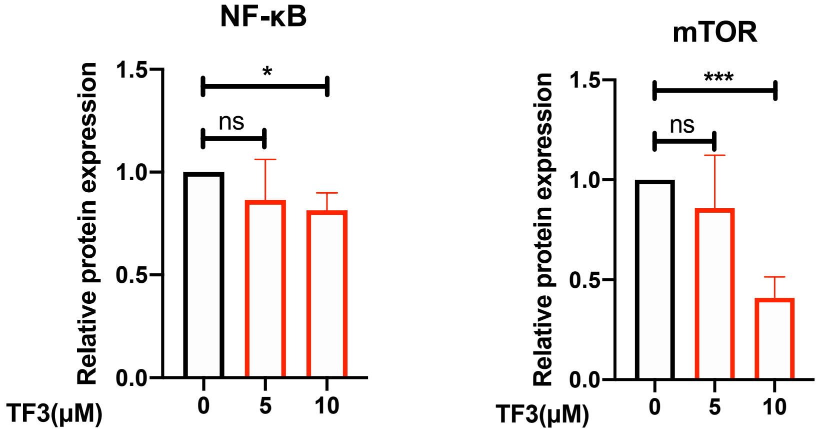

Supplementary Figure S2. Statistical analysis of NF-κB and mTOR protein expression in different groups. Granulosa cells were cultured for 24 h, starved in serum-free culture medium for 24h, and then TF3 (5μM,10 μM) or control treatment was given. The related

marker proteins were measured, and the statistical analysis was performed. ns, no significant difference; *p<0.05 and ***p<0.001.

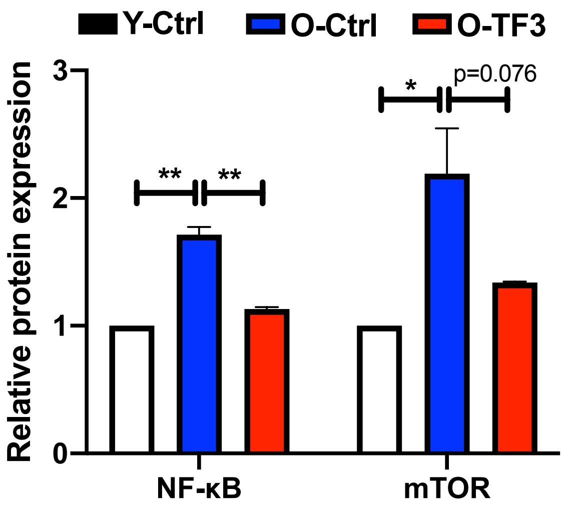

Supplementary Figure S3. Statistical analysis of NF-κB and mTOR protein expression in different groups. Ovarian tissues were collected from Y-Ctrl, O-Ctrl and O-TF3 group. The proteins were extracted to analyze the effects of TF3 on NF-κB and mTOR protein levels. The statistical analysis was performed. *p<0.05 and **p<0.01.
